# Investigating the relationship between satisfaction of basic psychological needs, general health, and some background variables in the Iranian older adults: a cross-sectional study

**DOI:** 10.1186/s12888-022-03979-z

**Published:** 2022-06-01

**Authors:** Hassan vismoradi ‑Aineh, Abbas Alipour, Ali Ramezankhani, Jalal Shakeri, Soudabeh Yarmohammadi, Tayebeh Marashi

**Affiliations:** 1 School of Public Health and Safety, Shahid Beheshti University of Medical Sciences, Tehran, Iran; 2 Community Medicine Department, Medical Faculty, Mazandaran University of Medical Sciences, Sari, Iran; 3 Department of Public Health, School of Public Health and Safety, Shahid Beheshti University of Medical Sciences, Tehran, Iran; 4 Department of Psychiatry, Substance Abuse Prevention Research Center, Health Institute, Kermanshah University of Medical Sciences, Kermanshah, Iran

**Keywords:** Psychological needs, Mental health, Older adults, Autonomy, Competence

## Abstract

**Background:**

Promoting the health and mental health (MH) of the older adults making up a large part of the world’s population in the coming years can provide the necessary conditions for their health and well-being of them. This study aimed to investigate the relationship between the satisfaction of basic psychological needs (BPNs), general health (GH), and some variables in Iranian older adults.

**Methods:**

The present descriptive-correlational study was conducted on 780 older adults from Sarpol-e Zahab (Kermanshah) in 2019 including the study by multi-stage cluster random sampling. The data collection tool was BPNs satisfaction and GH questionnaire and a researcher-made questionnaire of individual and background information. Was used for data analysis using the SPSS version 16 program and descriptive statistics and tests Pearson correlation coefficient, chi-square test, independent-sample T-test, and multivariate linear regression.

**Results:**

In the present study, participating a total of 780 older adult men aged 73.0 ± 29.32 years. There was a significant relationship between the satisfaction of BPNs and GH (*p* <  0.001). Also, 41% of the older adults were in poor GH and 30% were high in BPNs. Multiple logistic regression showed that the BPNs, age, income satisfaction, weather, and war zone were strong predictors of GH. the adjusted R2 value of 0.55 shows that the model described 55% of changes in the GH score.

**Conclusion:**

According to the findings of the study on the relationship between the satisfaction of BPNs and GH, providing insurance, social and economic support by developing health policies, creating supportive health environments, strengthening community action, and developing individual skills in the older adults can help improve their MH and that of the community.

## Background

Today, for the first time in human history, the life expectancy of many countries is 60 years or more. In low- and middle-income countries, this increase in life expectancy has been due to a large reduction in mortality, especially during childbirth, childhood, and death due to infectious diseases, and in high-income countries due to reduced mortality in the older adults [1].

According to the census of 1996 and 2001, people aged 60 and older in Iran were 6.62 and 7.27%, and in 2011, people aged 60 and older were equal to 8.26%. It is expected that in 2031, about 25–30% of the population of Iran will be 50 years and older [2]. The United Nations estimates that the population over the age of 65 will increase from one in 11 people in 2019 to six in 11 people in 2050 [3].

Aging is inevitable for humans. Aging, which is caused by genetic and environmental factors, is progressive and irreversible causing disability in the body without exception. This aging process in older adults is associated with diseases that mainly include chronic non-communicable diseases, cancer, and MH disorders that accelerate the body’s disability [4, 5].

Healthy aging is defined as the ability to lead a healthy lifestyle and be relatively free from disease or disability [6], and this is more likely in those who actively participate in activities to improve their health and well-being [7]. By raising the health level of the older adults, the GH of the community also increases. For human beings to be satisfied with life, it is necessary to meet a set of physical, social, and psychological needs [8].

One of the important components of quality of life in individuals is MH [9]. Economic problems, physical illnesses, disability, isolation due to retirement, and lack of proper community approach to the older adults can somehow reduce the social value and strengthen the incidence of psychological problems [10]. Satisfaction of psychological needs is a strong predictor of MH and is the basis for experiencing happiness and health, leading to the well-being of society [11].

According to the self-determination theory, the three basic needs of the psychological field that must be met are autonomy, competence, and relatedness [12]. Autonomy refers to a person’s desire to freely pursue his activities and the role of the individual’s will in doing work. Competence means the ability to perform tasks and to what extent the individual’s ability plays a role in achieving the desired goals. A relatedness is a form of social impact indicating a sense of belonging to those who are important to the individual [13].

Social participation is a determinant of active aging [14] and there is ample evidence that social participation can have positive consequences for both physical [15] and MH [16]. Social support and communication networks through social participation provide information that helps participants make better health and medical choices, so they have a healthier lifestyle [17].

Social participation provides positive psychological states, such as high self-esteem, a sense of belonging, and purpose in life, and has a positive effect on MH [15]. Social participation also has a protective effect against reduced physical function [18]. In addition, studies have shown that there is a relationship between loss of autonomy, deteriorating health, hospitalization, the onset of depressive symptoms, and apathy [19]. People feel competent when they can participate in experiences and activities in which they use their skills and expertise [20]. The older adults’ sense of competence is affected by changes in cognitive competencies as well as physical limitations and injuries [21]. Accordingly, meeting BPNs, such as autonomy, competence, and relatedness can help reduce mental diseases, such as depression and anxiety, and mobility reduction in older adults.

Psychological problems can be studied from various aspects, including ethnicity and race. Obstacles, such as differences in language and special beliefs in the older adults of specific races and ethnicities can make it difficult to diagnose and interpret psychological disorders. One of the ethnic groups living in Iran is the Kurds, who are the fifth ethnic group in the country in terms of population. Observance of age hierarchy and respect for the older adults are among the ancient customs among them. The Kurdish older adults have special social conditions due to the wars in the early years after Iran’s revolution and the 8-year war imposed by Iraq, which undoubtedly affected their MH [22]. War and displacement are among the destructive factors affecting MH [23]. Another factor affecting human MH has been the impact of climate [24]. The importance of environmental risk factors on MH outcomes has been considered [25]. Based on the evidence, exposure to extreme heat has negative effects on MH [26].

According to studies conducted in different parts of the world, older adults have high levels of mental disorders, indicating the need for careful assessment of MH and BPNs [27–31]. Moreover, studies on the self-determination theory and the older adults in nursing homes or hospitals have further emphasized the relationship between the three BPNs, well-being, and depression [32, 33]. However, according to the studies, none of the studies aimed to identify the relationship between BPNs and GH and individual and background factors (climate and war zone) in older adults.

Mental disorders are important in both sexes, but considering that in the Sarpol-e Zahab region (one of the Kurdish and war-torn regions of Iran) men play a major role in providing family income, any physical and mental disorders in addition to threatening the individual’s health, seriously affects the health of the family. Therefore, due to the importance of the subject and limited research in this regard, this study was conducted to investigate the relationship between the satisfaction of BPNs, GH, and some variables in male older adults in Sarpol-e Zahab.

## Methods

### Study area, design, and population

The present descriptive-correlational study was conducted in 2019 on the older adults of Sarpol-e Zahab city of Kermanshah. The older adults were selected from health centers. According to the statistics of the health center of this city in 2019, it had a population of more than 88 thousand people, of which 9054 people are over 60 years old. One thousand three hundred four older adults women and 1280 older adults men live in urban areas and 3515 older adults women and 2913 older adults men live in rural areas. Currently, the integrated care program at the city level is being implemented by 10 urban and rural centers and 50 health centers along with four community health centers some of these care include mental health. The study population is older adults men in Sarpol-e Zahab city.

### Inclusion and exclusion criteria

The inclusion criteria consisted of men aged 60 years and older, satisfaction with cooperation, living in Sarpol-e-Zahab city for at least 1 year, and no mental problems approved by a doctor. The exclusion criteria were unwillingness to cooperate and incomplete questionnaires.

### Sample size estimation and sampling procedure

This study aimed to investigate the relationship between the BPNs, GH, and some variables in older adults. The study population was older adult men in Sarpol-e-Zahab city. The sample size was estimated based on the following formula with Z = 1.96, *P* = 0.5, and d = 0.05.

$${\boldsymbol{N}}_{\mathbf{1}}=\frac{{\boldsymbol{Z}}^{\mathbf{2}}{}_{\mathbf{1}-\propto /\mathbf{2}}{} \ast \boldsymbol{P}\ast \left(\mathbf{1}-\boldsymbol{P}\right)}{{\boldsymbol{d}}^{\mathbf{2}}}$$

The sample size was based on a 95% confidence level, and 5% precision, and the probability of prevalence of mental disorders was about 50% equal to 385 older adults. Due to cluster sampling and considering the design effect equal to 1.7 to 2, the number of samples was estimated at 780 older adults.

Using multi-stage cluster sampling, 780 older adults were selected. First, urban and rural areas were selected as the first class (stratified sampling), then health centers with warm or temperate climates were selected as the second class (stratified sampling). Then, by cluster sampling method and based on the population share of the defined classes of health centers, half of the male older adults of those centers have randomly entered the study (based on the census list in the centers) in two subgroups of 60–75 years and over 75 years. The self-report questionnaires were completed after the written consent of the older adults.

### Data collection and procedures

#### Instrumentation

The questionnaires used in this study included the General Health Questionnaire (GHQ-28), Basic Psychological Needs Satisfaction Questionnaire (BNSQ), and a Researcher-made questionnaire of personal and background information.

#### General health questionnaire (GHQ-28)

This questionnaire was first designed by Goldberg in 1972, and in this study, its 28-item form was used, identifying discomfort in less than a month. It has 4 subscales, including physical symptoms, anxiety, sleep disorder symptoms, social functioning, and depressive symptoms. Each subscale has 7 questions and each of the four domains is given a score and the whole questionnaire (28 questions) is given a score. Thus, this scale gives 5 separate scores. In terms of answering the questions, the subject should complete the questionnaire using a four-point Likert scale (0, 1, 2, 3) according to their health status during the past month. On each scale, score 6 and above, and in total score 22 and above indicate pathological symptoms. Scores in the subscales and the whole questionnaire are “none or minimum (0-6), (0-22)”, “mild (7-11), (40-23)”, “average (16-12), (60-41)”, “severe (21-17), (84-61)” [34]. In Likert scoring, the maximum score is 84 and the cut-off point is 23. The translation, validity, and reliability of this questionnaire have been confirmed by Iranian researchers [35].

#### Basic psychological Need Satisfaction Questionnaire (BNSQ)

The questionnaire was designed by Guardian, Desi, and Ryan (2000) to measure the sense of support for the needs of autonomy, competence, and relatedness. The questionnaire consists of 21 questions scored on a 7-point Likert scale from absolutely true to not at all true [12]. A higher score in each area indicates a more favorable status. The minimum and maximum scores of the questionnaire are 21 and 147, respectively. Scores of 21 to 42 indicate low BPNs, scores of 42 to 105 indicate moderate BPNs, and scores above 105 indicate high BPNs. The translation, validity, and reliability of this questionnaire have been confirmed by Iranian researchers [36, 37].

#### Personal and background information questionnaire

This questionnaire includes 11 questions examining the personal and background information of the older adults (age, income satisfaction, place of residence, marital status, type of life) as well as the type of climate (temperate and warm) and the war zone.

### Statistical analysis

The data were entered into SPSS version 16 for statistical analysis. Chi-square, independent t-test, and Pearson correlation coefficient were used. The normality of the data was assessed by the Kolmogorov-Smirnov test and the significance level was less than 0.05 in the tests. Multiple linear regression was used to test the effect of BPNs and personal and background information variables on GH. GH was used as the dependent variable and BPNs and personal and background information variables as the independent variables.

## Results

The present descriptive correlational study was performed on 780 older adults men aged 60 to over 70 years. The mean age of the older adults was 73.0 ± 29.32 years. Moreover, 85.64% of the participants were married, 96% were with family and other personal and background information variables are described in Table 1. The mean scores of BPNs and GH and their subscales are shown in Table 2.

**Table 1:** Participants’ personal and background information in the research

Variables	Sub-group	Total sample N(780)
Marital status	Married	660(84.6%)
	Single	4(0.5%)
	Divorced	116(14.9%)
Income satisfaction	Yes	234 (30%)
	No	242 (31%)
	Somewhat	304 (39%)
Place of residence	Village	390 (50%)
	City	390 (50%)
Type of living	Living with family	738(94.62%)
	Living with a non-family	42(5.38%)
Climate	Warm	648(83.1%)
	Temperate	132(16.9%)
War zone	Yes	624 (80%)
	No	156 (20%)
Age	Mean ± SD	73.0 ± 29.32

**Table 2:** The mean scores of variables

Variable		Mean ± SD
Basic Psychological Needs		98.12 ± 16.53
	Autonomy	33.72 ± 6.84
	Competence	23.63 ± 6.27
	Relationship	40.76 ± 7.65
General health		46.78 ± 8.79
	Somatic Symptoms	12 ± 3.34
	Anxiety And Sleep Disorder	11.75 ± 3.17
	Social Function	12.91 ± 2.69
	Depression Symptoms	10.11 ± 2.87

The results showed that 41% of the older adults were in poor health status, 30% were high in BPNs, and 70% were at a moderate level. The correlation between the composite score of GH status (healthy and disorder) and the composite score of BPNs (low, moderate, and high) are shown in Table 3, which there was a statistically significant relationship between them (*p* <  0.001). The percentage of people with high BPNs in people with normal GH is more than people with a GH disorder.

**Table 3:** Relationship of basic psychological needs with general health

Variable	Subgroup	Basic psychological Needs			Total	*P*
		Low	Moderate)546)	High(234)		
		F^*^(%)	F(%)	F(%)	F(%)	
General health	normal	0	271(56.74)	197(43.26)	468(100)	<.001
	adverse	0	275(88.57)	37(11.43)	312(100)	
Total	–	0	546(69.82)	234(30.18)	780(100)	

F^*^ Frequency

The correlation between personal and background information and the composite score of BPNs is shown in Table 4. There was a significant relationship between the composite score of BPNs and the variables of age, climate, and income satisfaction (*P* <  0.001). Also, the Chi-square test did not show a significant relationship between the composite score of BPNs and place of residence, marital status, living status, and war zone (*P* > .05).

**Table 4:** Relationship of personal and background information variables with basic psychological needs

Variable	Subgroup	Basic Psychological Needs			*P*
		Low(*n* = 0)	Moderate(*n* = 546)	High(*n* = 234)	
^a^Age (y)	–	0	74.08(8.75)	71.06(8.38)	<.001
^b^Climate	Temperate	0	105(79.55)	27(20.45)	.002
	Warm	0	441(68.9)	207(31.1)	
^b^Income satisfaction	Yes	0	125(50.31)	109(49.69)	<.001
	No	0	204(85.99)	38(14.01)	
	Somewhat	0	217(71.59)	87(28.41)	

^a^Mean (SD)

^b^Frequency (%)

The correlation between personal and background information and the combined score of GH status is shown in Table 5. There was a significant relationship between GH and age, climate, income satisfaction, living conditions, and war zone (*P* <  0.001). In addition, the Chi-square test did not show a significant relationship between the combined score of GH status, place of residence, and marital status (*P* > 0.05).

**Table 5:** Relationship of personal and background information variables with general health

Variable	Subgroup	General Health		P
		Normal(*n* = 468)	Adverse(*n* = 312)	
^a^Age (y)	–	72.24(8.06)	70.04(7.68)	<.001
^b^Climate	Temperate	93(70.45)	39(29.55)	.002
	Warm	375(57.83)	273(42.17)	
^b^Income satisfaction	Yes	167(71.04)	67(28.96)	<.001
	No	114(46.25)	128(53.75)	
	Somewhat	187(59.88)	117(40.12)	
^b^Type of living	Living with a non-family	10(48.81)	14(51.19)	.02
	Living with family	458(59.62)	298(40.38)	
^b^War zone	Yes	423(67.15)	207(32.85)	<.001
	No	45(29.12)	105(70.88)	

^a^Mean (SD)

^b^Frequency (%)

### Predictors of general health

A multivariate linear regression analysis was used to test the effect of BPNs and personal and background information variables on GH. The dependent variable of GH and BPNs and personal and background information were independent variables. As shown in Table 6, The BPNs and age, income satisfaction, weather, and war zone were strong predictors of GH. the adjusted R2 value of 0.55 shows that the model described 55% of changes in the GH score.

**Table 6:** Multivariate regression analysis of the predictors of general health in the basic psychological need and personal and background information variables

Variables	95.0% confidence Interval for B		S	*p* value
	(Lower- upper bound)	Beta		
BPNs	(.22 _ .33)	.28	.025	<.001
Age	(.172 _ .35)	.26	.045	<.001
Climate	(−4.77 _ -.54)	−2.64	1.07	.01
war zone	(−9.77 _ -5.61)	−7.69	1.06	<.001
Income satisfaction				
Yes	(−4.41 _ -.29)	−2.35	1.05	.03
Some what	(−5.11 _ -2.39)	2.25	.95	<.001
Adjusted R Square = .55				

*BPNs* basic psychological needs, *S* standard error estimation

## Discussion

The current study aimed to investigate the correlation between the satisfaction of BPNs, GH, and some affecting variables in the older adults. The results showed that there was a significant relationship between the composite score of BPNs and the composite score of GH status. The percentage of people with high BPNs in people with normal GH was more than people with GH disorders. These findings can be explained by the fact that the satisfaction of BPNs has a positive effect on motivational variables, including intrinsic motivation. In other words, if the needs are satisfactorily met, people will be effectively involved in activities and achieve positive performance. The energy from satisfying psychological needs empowers the personality and the individual spontaneously engages in activities that increase MH [20].

The study by Behzadnia et al. [38] showed that illness and depression are a function of BPNs frustration. In the study by Okun et al. [39], it was reported that low psychological basic need causes poor sleep quality. Li et al. [40] reported that the satisfaction of psychological needs is negatively associated with stress and anxiety.

The present study showed that the GH in half of the older adults was poor. One of the reasons for the increase in GH disorders could be aging, which was also reported in the present study. According to Erikson’s stages of psychosocial development and Lazarus and Folkman’s transactional theory of stress and coping, significant differences in stressors are found at different ages [41, 42]. In the study of Luo et al. [43], the deterioration of the quality of life of the older adults related to health (physical and mental) is partly related to the biological weakness caused by their aging. To reduce the possible quality of mental life of the older adults, which may complicate health, prevention, and care of chronic illness and other illnesses, interventions should be made for high-risk individuals, including middle-aged people, before reaching old age.

In the present study, the total score of BPNs predicted the GH of older adults. Kouros et al. [44] reported that higher levels of autonomy predict lower levels of symptoms of failure and social anxiety among male helicopter parenting. Also, the results of the study by Ng et al. [45] showed that BPNs predict moderate to strong levels of patient well-being Including better mental health and higher levels of health behaviors (physical activity and consumption of prescription drugs) that are related to physical health and longevity.

Income inequality in a society is a determining factor in population health and there is an argument that socio-economic conditions affect health through psychosocial health [46]. According to the present study, income is another factor that has affected the GH of older adults. Also, income satisfaction is also determined as a strong predictor of GH. So people with GH disorders are not satisfied with their income. Studies in high-income countries have shown that improvements in MH and well-being are achieved after retirement (due to good payment) [47, 48]. Boutayeb et al. [49] also reported that there was a relationship between low income and unemployment with the prevalence of several diseases. There is a close relationship between job loss and symptoms of common mental disorders, such as depression and anxiety [50]. The study also showed that the older adults with high BPNs had higher income satisfaction. Di Domenico et al. [51] reported that there was a relationship between higher levels of income inequality and lower levels of estimation of BPNs.

In the present study, the older adults living with families had good GH, but a significant number also reported GH disorders. The older adults who lived with their family but had a GH disorder which could be due to some reasons. Their spouse may have died, they may be living with their children, and they may not have a source of income and their responsibility is with those who they live with, or they have physical and mental problems and it is difficult to keep them at home, which can cause problems for the older adults at home. This issue requires further research. In the study by Drageset et al. [52] and Tiong et al. [53], the home loss was one of the factors that had an adverse effect on the MH of the older adults in nursing homes. The results of these studies are not in line with the present study, which might be due to the fact that these studies included both genders in their study, while in the present study, only male older adults participated. It is possible that the men in this study had GH disorders due to low BPNs and a lack of independence and competence in their families.

Some studies have reported the risks of a wide range of MH-related consequences with high temperatures [54]. The present study showed that the older adults living in warmer climates had more GH disorders than those living in temperate regions. Also in the present study, it was reported that weather is a predictor of GH. In the study by Li et al. [55], it was reported that by increasing temperature, hospitalization in the emergency department due to anxiety, depression, and mental disorders increased. Gao et al. [56] stated that personal characteristics and contextual factors, such as age, gender, socio-economic factors, and ambient temperature lead to greater vulnerability in individuals.

As shown in the present study, the older adults who lived in war zones had GH disorders and war is a predictor of GH. Children who spent their childhood during the war had a reduced ability to regulate stress and fear responses at later ages, thus increasing their risk of developing mental and behavioral disorders in adulthood [57]. The study by Newnham et al. [58] also reported that psychological trauma increases for populations remaining in the post-war environment.

Accordingly, implementing strategies, such as developing health policies, creating health support environments, strengthening community action, developing individual skills, and nationwide reviewing of health care services providers, are likely to have a significant impact on reducing MH inequalities. There will be the greatest potential for achieving a healthy population, which includes reducing poverty, lifelong social support, reducing inequality, preventing war and conflict, and promoting access to employment.

## Limitations

This study had several limitations. First, this study examined the BPNs and GH as a total score. In order to better understand which BPNs affect the GH of the older adults, income satisfaction, and war experience, it is required to separately examine the relationship of each of the subscales of this need with other variables. Another limitation was that this study was a correlational study and could not infer causal results from it, since each of the variables could have a causal effect on the other or there might be a third variable that has a causal effect on the two variables. In future studies, it is suggested that experimental or longitudinal studies be conducted on the effectiveness of satisfying psychological needs on the GH of the older adults, which can show the causal relationship between the two variables. Moreover, stronger statistical methods, such as structural equation modeling, can be used to show the multivariate relationship between different variables related to GH and psychological needs and other variables.

## Conclusion

The present study aimed to find factors related to the GH of the older adults. The results indicated that BPNs are related to GH and the more limited and obstructed the satisfaction of BPNs (autonomy, competence, and relatedness), the more the GH of the elderly decreases. Due to the war experience, unemployment, economic and social problems while living in a border town, such as Sarpol-e Zahab (Kermanshah), the older adults are less able to experience the satisfaction of BPNs and as a result, have less MH. The findings of this study can also provide a way to conduct research in this area playing an important role in promoting the health of the older adults. The self-determination theory provides a good framework for psychological and social issues. Most importantly, the results of the present study may have important implications for designing effective interventions.

## Data Availability

The datasets used and/or analyzed during the current study are available from the corresponding author on reasonable request.

## Abbreviations

BPNs: Basic Psychological Needs
GH: General Health
MH: Mental Health
